# Near-infrared emitting CdTe_0.5_Se_0.5_/Cd_0.5_Zn_0.5_S quantum dots: synthesis and bright luminescence

**DOI:** 10.1186/1556-276X-7-615

**Published:** 2012-11-06

**Authors:** Ping Yang, Shiquan Wang, Norio Murase

**Affiliations:** 1 Health Research Institute, National Institute of Advanced Industrial Science & Technology (AIST), Hayashi-cho, Takamatsu-city, Kagawa, 761-0395, Japan; 2 School of Material Science and Engineering, University of Jinan, Jinan, 250022, People’s Republic of China

**Keywords:** Quantum dots, Semiconductor, Photoluminescence, Near-infrared, Core/shell

## Abstract

We present how CdTe_0.5_Se_0.5_ cores can be coated with Cd_0.5_Zn_0.5_S shells at relatively low temperature (around 200°C) via facile synthesis using organic ammine ligands. The cores were firstly fabricated via a less toxic procedure using CdO, trioctylphosphine (TOP), Se, Te, and trioctylamine. The cores with small sizes (3.2-3.5 nm) revealed green and yellow photoluminescence (PL) and spherical morphologies. Hydrophobic core/shell CdTe_0.5_Se_0.5_/Cd_0.5_Zn_0.5_S quantum dots (QDs) with tunable PL between green and near-infrared (a maximum PL peak wavelength of 735 nm) were then created through a facile shell coating procedure using trioctylphosphine selenium with cadmium and zinc acetate. The QDs exhibited high PL efficiencies up to 50% because of the formation of a protective Cd_0.5_Zn_0.5_S shell on the CdTe_0.5_Se_0.5_ core, even though the PL efficiency of the cores is low (≤1%). Namely, the slow growth process of the shell plays an important role for getting high PL efficiencies. The properties of the QDs are largely determined by the properties of CdTe_0.5_Se_0.5_ cores and shells preparation conditions such as reaction temperature and time. The core/shell QDs exhibited a small size diameter. For example, the average diameter of the QDs with a PL peak wavelength of 735 nm is 6.1 nm. Small size and tunable bright PL makes the QDs utilizable as bioprobes because the size of QD-based bioprobes is considered as the major limitation for their broad applications in biological imaging.

## Background

Semiconductor quantum dots (QDs) have attracted considerable interest in the past decade because of their excellent properties such as narrow, symmetric, and tunable emission spectra, broad absorption spectra, superior photostability, high photoluminescence (PL) efficiency, and the capacity of simultaneous excitation of multiple fluorescence colors compared with the organic dyes and fluorescent proteins [1-4]. Moreover, the near-infrared (NIR) spectral range presents many advantages for *in vivo* fluorescence imaging. The absorption of photons by biological tissues is much reduced in this spectral window compared with the visible range. There are many more alternatives in QDs with NIR emission for *in vivo* imaging than organic dyes [5]. For example, the QDs with a PL of 700-nm region minimize the problems of indigenous fluorescence of tissues and meet the requirements of *in vivo* biological imaging applications. Therefore, the synthesis of NIR-emitting QDs (700–900 nm) has received much attention recently. In addition, the QDs have advantages compared with metallic nanoparticles for nanoprobes because of their broad absorption band and tunable and bright PL [6].

CdSe is still one of the most popular QDs created by various chemical strategies. However, CdSe (*E*_g_ = 1.76 eV, *a*_B_ = 9.6 nm) QDs can be tuned through quantum confinement to emit visible fluorescent spectrum only reaching typically 650–700 nm. Furthermore, it is difficult to grow CdSe-based QDs with NIR emission because of their growth kinetics. In addition, the size of CdSe with a longer PL peak wavelength becomes too large for bioprobers. Compared with CdSe, the band gap of bulk CdTe is narrow (1.56 eV, corresponding to approximately 795 nm wavelength). The PL peak wavelength of CdTe QDs with a diameter of 7 nm can reach approximately 720 nm. However, CdTe and CdTe/CdSe QDs prepared by an organic synthesis are normally unstable, which results in low PL efficiency and fast photobleaching. Therefore, composited CdTe and CdSe QDs or CdTe-based QDs with protective inorganic shells are expected. Especially, these QDs with NIR emission and small size are necessary. However, there have been few reports of a higher band gap shell growth around CdTe or CdTeSe QDs. Rogach and co-workers described the aqueous synthesis of 8–10 nm CdTe-based QDs emitting at approximately 800 nm [7].

For a purpose of getting a highly stable fabricating CdTeSe-composited cores with a higher band gap shell is still a challenge. Nanoalloying of two semiconductors shows properties distinct not only from the properties of their bulk counterparts (such as CdTe_0.5_Se_0.5_), but also from those of their parent semiconductors (such as CdTe or CdSe). Engineering the band gap energy of QDs has resulted in the development of core/shell QDs with novel exciting properties and applications. Recently, Jiang et al. reported that they were able to deposit only a very thin CdS shell (approximately one monolayer) on the surface of a CdTeSe core [8]. However, the band gap of CdS is not large enough to provide the potential barrier necessary to block both electrons and holes inside CdTeSe cores. A ZnS shell has a crucial role in the improvement of PL properties of QDs. This increases the chemical stability and photostability of the QDs because ZnS is a chemically stable with wide band gap (3.8 eV for the bulk material) semiconductor material. However, the large mismatch between CdTeSe and ZnS lattice parameters enables the strain at the interface between the core and the shell. Therefore, a Cd_*x*_Zn_1−*x*_S composite shell can also improve the stability and PL properties of the QDs. Very recently, we fabricated CdSe cores coated with Cd_*x*_Zn_1−*x*_S composite shells [9]. These core/shell QDs reveal broadly tunable PL spectra (PL peak wavelength up to 650 nm), narrow size distribution, high PL efficiency (up to 60%, in the case of CdSe cores with a PL efficiency of 1%), and a high stability (keeping their initial PL efficiency in a toluene solution for 1 year).

To reduce the lattice parameter mismatch and improve the properties of core/shell QDs, an interlayer is inserted, such as CdS and ZnSe between the CdSe core and ZnS shell. A Cd_*x*_Zn_1−*x*_S composite shell has led to the formation of CdS interlayer due to the growth kinetics of CdS and ZnS. Thus, CdTeSe cores would strongly benefit from Cd_*x*_Zn_1−*x*_S composite shells for getting a longer PL peak wavelength, as well as high stability and PL efficiency. However, very little reports focused on the NIR emission of composite cores coated with a higher band gap composited shell. Recently, Pons et al. reported that near-infrared-emitting CdTeSe/CdZnS core/shell QDs prepared using cadmium oxide (CdO), zinc oxide (ZnO), tetradecylphosphonic acid (TDPA), cadmium oleate (Cd(OA)_2_), and zinc oleate (Zn(OA)_2_) as precursors revealed a high stability [10]. Because of decreased lattice mismatch between the core and the shell, the interface strain decreases dramatically during the shell growth, indicating that the spatial distribution of defects in core/shell QDs can be controlled by the shell growth. A better understanding of the shell effect on optical properties of QDs is important for optimizing the synthesis. Because Cd-based QD is undesirable for biological imaging due to toxicity, a CdZnS shell with a ZnS-rich layer coated on the surface of CdSe is used to decrease the release of Cd ions in this paper. We further have a technique of glass coating on the surface of the QDs as well for the purpose of drastically reducing the amount of Cd released from the QDs as demonstrated in the previous publications [11,12].

For the preparation of QDs by classical colloidal chemistry, organometallic and/or metal organic compounds under anaerobic conditions have been employed. For example, CdSe QDs were prepared by reacting dimethylcadmium (CdMe_2_) with trioctylphosphine selenium (TOP)Se in TOP/trioctylphosphine oxide or TOPO at high temperature [13]. Therefore, a novel and facile synthesizing strategy, which uses a faster reaction process and less toxic chemicals, is highly desirable. To address a novel method for fabricating QDs with NIR PL, more efforts to other potential preparation methods of QDs have been taken in our group. In this paper, CdTe_0.5_Se_0.5_/Cd_0.5_Zn_0.5_S core/shell QDs were fabricated using less toxic chemicals and a facile synthesis. These core/shell QDs revealed tunable PL peak wavelength from the initial value of CdTe_0.5_Se_0.5_ cores to 735 nm with much improved PL efficiency above 50%. A detailed consideration allows us to grow multilayer Cd_0.5_Zn_0.5_S shell around CdTe_0.5_Se_0.5_ QDs using air stable zinc and cadmium acetate precursors at relatively low temperature (approximately 200°C). The resulting core/shell QDs exhibit improved stability and PL efficiency compared with CdTe/Cd_0.5_Zn_0.5_S QDs.

## Methods

### Chemicals

All chemicals were of analytical grade or of the highest purity available and used directly without any further purification. Cadmium oxide (CdO, 99.99%), cadmium acetate dihydrate (Cd(Ac)_2_2H_2_O, 98%), zinc acetate (Zn(Ac)_2_, 99.99%), selenium (Se, 99.5%), sulfur (S, 99.98%, powder), tellurium (Te, 99.99%), octadecylphosphonic acid (ODPA, 97%), trioctylamine (TOA, 98%), octadecene (ODE, technical grade of 90%, ODE), TOP (97%), hexadecylamine (HDA), and TOA were supplied by Sigma Aldrich (St. Louis, MO, USA). The pure water was obtained from a Milli-Q synthesis system (resistivity of 18.2 MΩ·cm) (EMD Millipore Corporation, Billerica, MA, USA).

### Preparation of CdTe_0.5_Se_0.5_, CdSe, and CdTe cores

All preparations were carried out under N_2_ atmosphere. A typical CdTe_0.5_Se_0.5_ core synthesis is composed of one-step growth as described hereafter. A 0.54 mmol of CdO and 180 mg of ODPA were mixed with 7 mL of TOA in a three-neck round-bottom flask under N_2_ flow, and stirred at 140°C for 1 h to remove adsorbed H_2_O. Then the mixture was kept at 280°C until the CdO completely dissolved. A 0.5 mmol of Se powder and Te powder were completely dissolved in 1 mL of TOP with stirring. The TOPSe with TOPTe solution was then swiftly injected into the cadmium precursor solution with stirring, and kept at 280°C for 2 min, followed by cooling down to room temperature. A 10 mL of hexane and 50 mL of ethanol were added to precipitate as-prepared samples. The resulting sample was then washed with copious ethanol, re-dispersed in 20 mL of toluene, and centrifuged at 12,000 rpm for 5 min to remove the sludge. Next the cores were precipitated with ethanol and re-dispersed in 10 mL of toluene for subsequent shell coating. To prepare CdTe_0.5_Se_0.5_ cores with various sizes, the reaction time and temperature as well as the injection speed of TOPSe and TOPTe were adjusted. The preparation conditions and properties of CdTe_0.5_Se_0.5_ cores and CdTe_0.5_Se_0.5_/Cd_0.5_Zn_0.5_S core/shell QDs are summarized in Table 1.

**Table 1:** **Preparation conditions and properties of CdTe **_**0.5**_**Se**_**0.5 **_**cores and CdTe**_**0.5**_**Se**_**0.5**_**/Cd**_**0.5**_**Zn**_**0.5**_**S core/shell QDs**

**Sample**	**Reaction time (min)**	**Reaction temperature (°C)**	**Composition**	**PL peak wavelength (nm)**	**PL efficiency (%)**	**FWHM (nm)**
1	2.5	280	CdTe_0.5_Se_0.5_	573.4	0.9	23.6
2^a^	6	220	CdTe_0.5_Se_0.5_/Cd_0.5_Zn_0.5_S	700.4	48.3	65.2
3	2.5	280	CdTe_0.5_Se_0.5_	576.4	1.1	38.4
4^a^	11	200	CdTe_0.5_Se_0.5_/Cd_0.5_Zn_0.5_S	656.2	52.2	64.4

^a^These samples were prepared by using samples 1 and 3, respectively.

Alternatively, CdSe cores were fabricated by injecting 1 mL of TOPSe with 1 mmol Se in the precursor of Cd within 30 s under N_2_ flow. After further reaction for 2 min, the resulting sample was separated by using a similar procedure with that of CdTe_0.5_Se_0.5_ cores. CdTe cores were synthesized by addition of 1 mL of TOPTe with 1 mmol Te within 30 s under N_2_ flow. The CdTe cores were precipitated twice in ethanol and resuspended in 10 mL of toluene.

### Preparation of CdTe_0.5_Se_0.5_ and CdTe cores coated with Cd_0.5_Zn_0.5_S shell

The preparation of Cd_0.5_Zn_0.5_S shell was carried out by directly reacting cadmium and zinc acetate with TOPS. In a typical synthesis, 14 mg of Cd(Ac)_2_2H_2_O, 10 mg of Zn(Ac)_2_, 0.5 g of HDA, and 5 mL of ODE were mixed in a three-neck round-bottom flask under N_2_ flow and stirred at 200°C until the Cd and Zn salts were completely dissolved. A 6 mg of S powder was dissolved in 0.5 mL of TOP. The toluene solution of CdTe_0.5_Se_0.5_ or CdTe cores (3 mL) was injected with vigorous stirring followed by the injection of the TOPS solution. The mixture was kept at 200°C with stirring for several to several 10 min, followed by cooling down to room temperature. The products were precipitated, washed twice with ethanol, and re-dispersed in 10 mL of toluene. To investigate the effect of preparation conditions on properties, reaction time and temperature were adjusted.

### Apparatus

The transmission electron microscopy (TEM) observations of samples were performed mainly using a Hitachi EF-1000 electron microscope (Hitachi Ltd., Tokyo, Japan). The absorption and PL spectra were recorded using conventional spectrometers (Hitachi U-4000 and F-4500, respectively). Both excitation and emission slits are 5 nm for the measurement of PL spectra. The PL efficiencies of samples in solution were estimated using a method previously reported [14]. Briefly, the PL and absorption spectra of a standard quinine solution (quinine in 0.1 N H_2_SO_4_ solution; PL efficiency *η*_0_ of 55%) were measured in a 1-cm quartz cell as a function of the concentration.

## Results and discussion

Alloyed semiconductor QDs can be classified as having whether (1) a uniform internal structure (i.e., homogeneous) or (2) a gradient internal structure, where the alloy composition varies in different parts of the QD [15,16]. The structure of these QDs differs from that of core/shell QDs, where a thin layer of a wider band gap semiconductor is grown on the surface of a core semiconductor. The properties of alloyed QDs are characterized by Végard’s law which states that while lattice constant changes linearly with composition, other physical properties such as band gap often vary nonlinearly. In the case of QDs of any composition, quantum confinement also dictates size dependence [17] as shown in this equation:

$$E_{g}\left(d\right)=E_{g}\left(\infty \right)+\frac{a}{d}+\frac{c}{d^{2}}\text{,}$$

where *d* is the QD diameter and *a* and *c* are empirical fit parameters. Since the PL properties of QDs depended strongly on their band gap and size, the alloyed CdTe_0.5_Se_0.5_ QDs revealed different PL properties compared with CdTe and CdSe QDs.

The quantum confinement of QDs with any composition dictates size dependence. Similar to bulk CdTe_*x*_Se_1−*x*_ alloy, a very slight nonlinear relationship between the band gap energy and the composition, known as ‘optical bowing’, was also observed for the QDs. For example, Han’s group reported similar observations for homogeneous Zn_*x*_Cd_1−*x*_S QDs. To investigate the effect of composition on PL properties, we prepared three kinds of QDs including CdTe_0.5_Se_0.5_, CdTe, and CdSe by using the same preparation procedure as indicated in ‘Methods’ section. Table 2 summarizes the property comparison of CdTe, CdSe, and CdTe_0.5_Se_0.5_ QDs. CdTe_0.5_Se_0.5_ QDs revealed a longer PL peak wavelength and smaller size than those of CdSe QDs. Compared with CdTe QDs, alloyed CdTe_0.5_Se_0.5_ QDs can provide better stability because of Se. In our experiment the PL of as-prepared CdTe QDs was quenched after 2 days. In contrast, both of CdSe and CdTe_0.5_Se_0.5_ QDs kept their initial PL efficiency after 1 month.

**Table 2:** **Property comparison of CdTe, CdSe, and CdTe**_**0.5**_**Se**_**0.5 **_**QDs**

**Composition**	**PL peak wavelength (nm)**	**Diameter (nm)**
CdTe	550	2.8
CdSe	544	4.0
CdTe_0.5_Se_0.5_	570	3.2

The size of QDs depended strongly on preparation conditions. To create CdTe_0.5_Se_0.5_ cores with various sizes, reaction time and the injection speed of TOPSe and TOPTe were adjusted. The PL spectra of CdTe_0.5_Se_0.5_ cores red-shifted with time under a low injection speed. This is ascribed to the reaction time and concentration dependence of precursor for the nucleation and growth of the cores. As summarized in Tables 1, 3, and 4, the PL peak wavelength of the cores was 573 to 592 nm.

**Table 3:** **Preparation condition and properties of CdTe**_**0.5**_**Se**_**0.5**_**/Cd**_**0.5**_**Zn**_**0.5**_**S QDs**

**Sample**	**Reaction time(min)**	**PL peak**	**PL efficiency**	**FWHM (nm)**
5^a^	N/A	588.2	0.9	21.4
5-1	1	589.4	36.2	44.2
5-2	2	603.4	44.2	56.4
5-3	3	611.0	46.2	62.2
5-4	5	614.4	48.9	67.4
5-5	7	621.6	52.5	67.4
5-6	10	630.4	51.6	67.6
5-7	15	643.8	43.0	71.6
5-8	20	663.4	44.4	77.0
5-9	35	683	38.9	86.0

^a^The core/shell QDs were prepared by using sample 5. The properties of CdTe_0.5_Se_0.5_ cores are listed for comparison.

*N/A* not available.

**Table 4:** **Preparation condition and properties of CdTe**_**0.5**_**Se**_**0.5**_**/Cd**_**0.5**_**Zn**_**0.5**_**S QDs**

**Sample**	**Reaction time(min)**	**PL peak**	**PL efficiency**	**FWHM (nm)**
6^a^	N/A	592.4	1.0	39.4
6-1	3	611.2	7.7	47.6
6-2	6	639.6	19.9	55.4
6-3	9	653.6	21.9	55.4
6-4	12	664.4	26.9	57.8
6-5	15	674.4	15.0	62.8
6-6	18	682.8	19.7	67.4
6-7	21	692.2	28.5	73.0
6-8	25	702.6	48.9	80.4
6-9	30	716.6	45.85	92.0
6-10	35	735.2	40.54	94.2

^a^The core/shell QDs were prepared by using sample 6. The properties of CdTe_0.5_Se_0.5_ cores are listed for comparison.

*N/A* not available.

Because CdSe/CdS QDs have less lattice mismatch (approximately 3.9%) than that of CdSe/ZnS QDs (approximately 12%) [18,19], an alloy Cd_*x*_Zn_1−*x*_S shell can provide better stability by decreasing interfacial strain between cores and shells. Quyang et al. reported that gradiently alloyed Zn_*x*_Cd_1−*x*_S QDs having a Cd-rich core and Zn-rich shell could be synthesized via a noninjection one-pot approach [20]. In our experiments CdTe_0.5_Se_0.5_ cores were coated with Cd_0.5_Zn_0.5_S composite shell by an organic one-step synthesis. The shell layers of CdS and ZnS might not be clearly separated, and it is also possible that the shell layer is a gradient alloy structure of CdS and ZnS. This is ascribed to the growth kinetic of CdS and ZnS. The gradient structure would be beneficial to improve stability by reducing lattice mismatch between CdSe and ZnS layers.

According to our previous experimental results, an optimal molar ratio of Cd/Zn of 1/1 was used for CdTe_0.5_Se_0.5_ cores coated with Cd_0.5_Zn_0.5_S shell for getting high PL efficiencies. To investigate the effect of temperature on the formation of Cd_0.5_Zn_0.5_S shell on CdTe_0.5_Se_0.5_ cores, reaction temperature was adjusted from 180°C to 280°C. An optimal temperature range was found to be around 190°C to 220°C. In the case of a high temperature (more than 260°C), the reaction solution became black quickly when TOPS was injected. This is ascribed to the PL quenching of QDs because the high temperature led to large amount defects generated during preparation. Table 1 summarizes the preparation conditions and properties of CdTe_0.5_Se_0.5_ cores and CdTe_0.5_Se_0.5_/Cd_0.5_Zn_0.5_S QDs.

**Figure 1:**
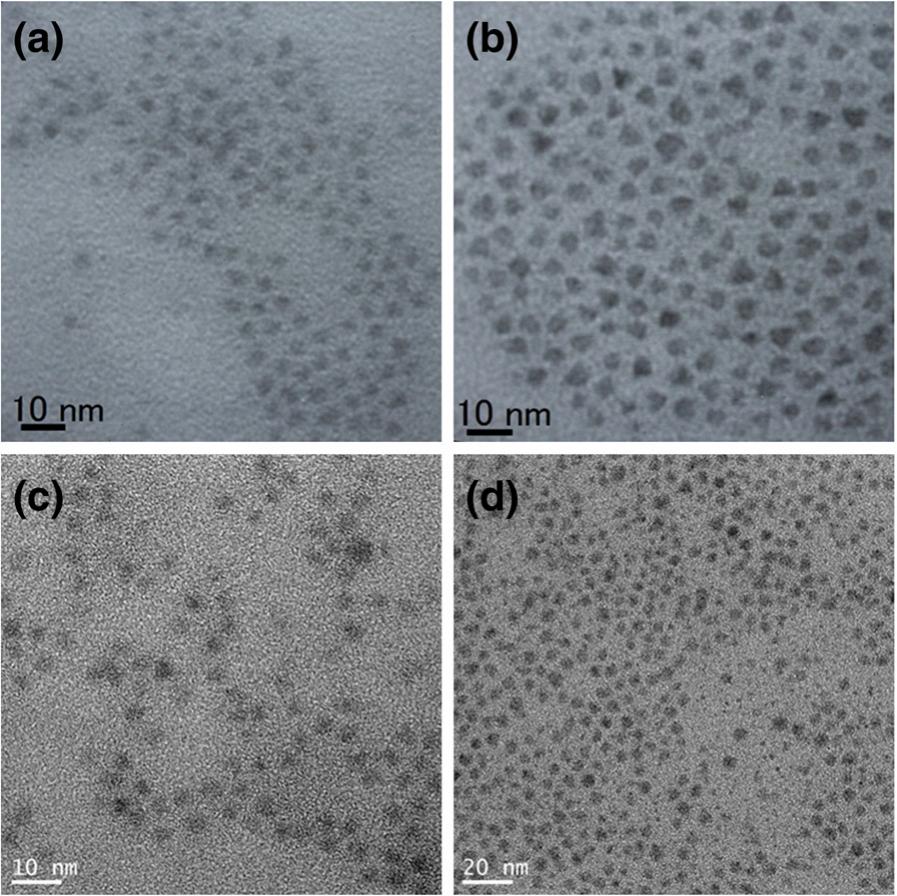
**TEM images of CdTe**_**0.5**_**Se**_**0.5**_**cores and CdTe**_**0.5**_**Se**_**0.5**_**/Cd**_**0.5**_**Zn**_**0.5**_**S core/shell QDs shown in Table**2**.** (**a**) Sample 1 (CdTe_0.5_Se_0.5_ cores), (**b**) sample 2 (CdTe_0.5_Se_0.5_/Cd_0.5_Zn_0.5_S) prepared by sample 1, (**c**) sample 3 (CdTe_0.5_Se_0.5_), and (**d**) sample 4 (CdTe_0.5_Se_0.5_/Cd_0.5_Zn_0.5_S) prepared by sample 3.

Figure 1 shows the TEM images of CdTe_0.5_Se_0.5_ cores and CdTe_0.5_Se_0.5_/Cd_0.5_Zn_0.5_S core/shell QDs shown in Table 1: (a) sample 1 (CdTe_0.5_Se_0.5_ cores), (b) sample 2 (CdTe_0.5_Se_0.5_/Cd_0.5_Zn_0.5_S) prepared by sample 1, (c) sample 3 (CdTe_0.5_Se_0.5_), and (d) sample 4 (CdTe_0.5_Se_0.5_/Cd_0.5_Zn_0.5_S) prepared by sample 3. Samples 2 and 4 were prepared at 220°C and 200°C, respectively. CdTe_0.5_Se_0.5_ cores revealed a spherical morphology (samples 1 and 3). In contrast, the core/shell QDs (samples 2 and 4) exhibited a tetrahedron-like morphology. This is ascribed to the CdS interlayer anisotropic deposition on CdTe_0.5_Se_0.5_ cores. For comparison, we fabricated CdTe_0.5_Se_0.5_/ZnS QDs using a similar preparation procedure. The result indicates that CdTe_0.5_Se_0.5_ coated with a ZnS shell revealed a spherical morphology. This indicates the anisotropic deposition of CdS on the core occurs.

**Figure 2:**
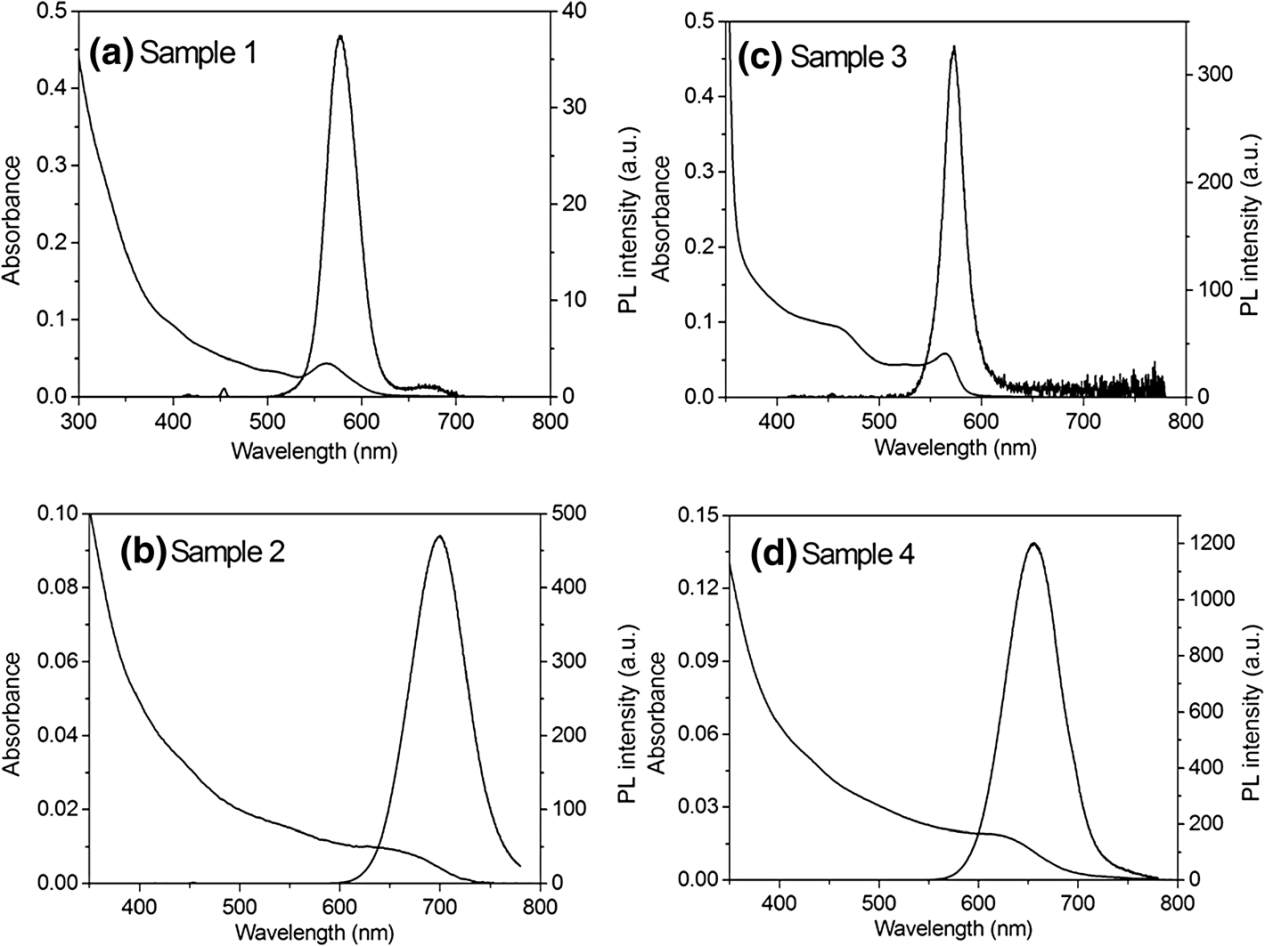
**Absorption and PL spectra of CdTe**_**0.5**_**Se**_**0.5**_**cores and CdTe**_**0.5**_**Se**_**0.5**_**/Cd**_**0.5**_**Zn**_**0.5**_**S core/shell QDs shown in Table**2**.** (**a**) Sample 1 (CdTe_0.5_Se_0.5_ cores), (**b**) sample 2 (CdTe_0.5_Se_0.5_/Cd_0.5_Zn_0.5_S Core/shell QDs), (**c**) sample 3 (CdTe_0.5_Se_0.5_/Cd_0.5_Zn_0.5_S core/shell QDs), and (**d**) sample 4 (CdTe_0.5_Se_0.5_/Cd_0.5_Zn_0.5_S Core/shell QDs). Samples 2 and 4 were prepared by using samples 1 and 3, respectively.

Figure 2 shows the absorption and PL spectra of CdTe_0.5_Se_0.5_ cores and core/shell CdTe_0.5_Se_0.5_/Cd_0.5_Zn_0.5_S QDs shown in Table 1: (a) sample 1 (CdTe_0.5_Se_0.5_ cores), (b) sample 2 (CdTe_0.5_Se_0.5_/Cd_0.5_Zn_0.5_S core/shell QDs), (c) sample 3 (CdTe_0.5_Se_0.5_/Cd_0.5_Zn_0.5_S core/shell QDs), and (d) sample 4 (CdTe_0.5_Se_0.5_/Cd_0.5_Zn_0.5_S core/shell QDs). A significant red shift was observed in both the absorption and PL spectra of the core/shell QDs in contrast to those of CdTe_0.5_Se_0.5_ cores. This is ascribed to the formation of a CdTe_0.5_Se_0.5_/Cd_0.5_Zn_0.5_S core/shell structure. Being coated with a Cd_0.5_Zn_0.5_S shell, the PL efficiency was drastically increased as shown in Table 1. However, the full width at half maximum (FWHM) of PL spectra was increased. This is because of the size distribution which became broad after coating with the shell.

For traditional QD synthesis by organic amine as a capping agent, researchers normally selected metal oleate and stearate as precursors. The combination of precursor and ligand is important for the non-coordinating solvent system. Metal stearate is a relatively stable precursor that could be coheated with a higher ratio of alkylamines such as 1-octadecylamine and HDA at high temperature (280°C-320°C) [20]. In contrast nucleation and growth in a metal oleate and HDA reaction system are executed at a moderate reaction temperature (265°C-285°C). In the present experiments, CdTe_0.5_Se_0.5_ cores were coated with Cd_0.5_Zn_0.5_S shells using cadmium and zinc acetate as precursors to react with TOPS in ODE with HDA. The reaction temperature can be further lowered because of salt acetate with a short carbon chain. In addition the preparation of the core/shell QDs has to be carried out quickly to prevent the decomposition of salt acetate. Namely, cadmium and zinc acetate can be decomposed into CdO and ZnO if the solution was kept at a high temperature (more than 240°C) for a long time (more than 20 min). In contrast the reaction for the formation of QDs can be easily controlled at low temperature (less than 210°C). Because of a slow growth procedure, the core/shell QDs revealed a high PL efficiency as shown in Table 1.

**Figure 3:**
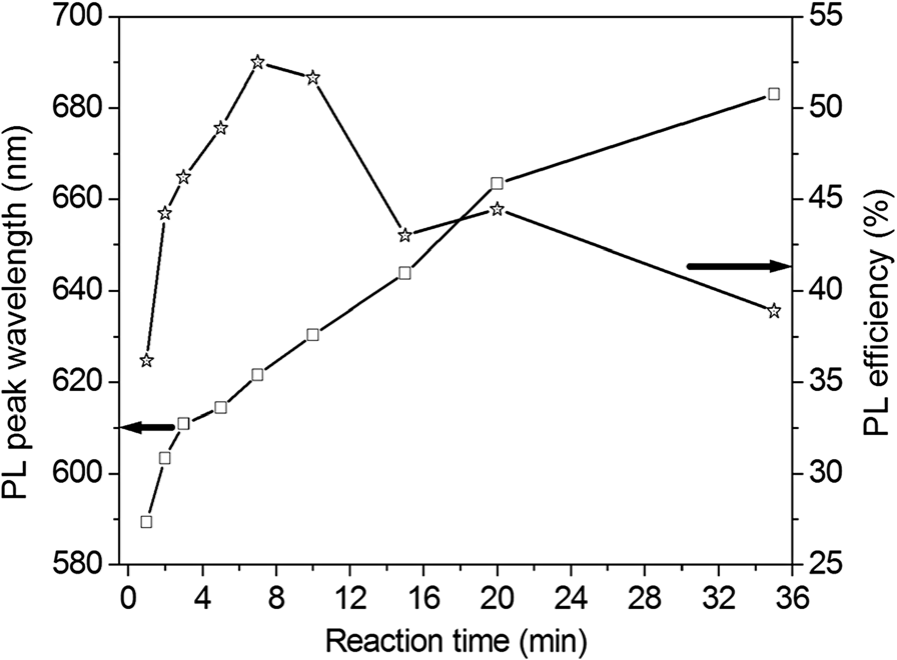
**PL peak wavelength and efficiency of CdTe**_**0.5**_**Se**_**0.5**_**/Cd**_**0.5**_**Zn**_**0.5**_**S core/shell QDs (Samples 5–1 to 5–9 shown in Table**3**) versus reaction time.** The core/shell QDs were prepared by sample 5. Star in the figure shows efficiency, while white square shows PL peak wavelength.

To clarify the effect of reaction time on PL properties of CdTe_0.5_Se_0.5_/Cd_0.5_Zn_0.5_S core/shell QDs, Table 3 summarizes the preparation condition and properties of CdTe_0.5_Se_0.5_/Cd_0.5_Zn_0.5_S QDs prepared by sample 5 (CdTe_0.5_Se_0.5_ cores). The PL properties of sample 5 are listed for comparison. Figure 3 shows the PL peak wavelength and efficiency of CdTe_0.5_Se_0.5_/Cd_0.5_Zn_0.5_S core/shell QDs (samples 5–1 to 5–9 shown in Table 3) versus reaction time. A gradual red shift of PL spectra was observed with time. This is ascribed to the thickness increase of the Cd_0.5_Zn_0.5_S shell with prolonged reaction time. The FWHM of PL spectra was increased after coating with a Cd_0.5_Zn_0.5_S shell. The formation of a Cd_0.5_Zn_0.5_S shell around CdTe_0.5_Se_0.5_ cores resulted in a drastic improvement of PL efficiency (up to 52.5 % when the reflux time is 7 min). The PL efficiency of the core/shell QDs was decreased with further increasing the reflux time. This is ascribed to the lattice mismatch generated by increasing shell thickness.

The PL efficiency of QDs is significantly influenced by the QDs’ surface state, such as the stoichiometric ratio of anion and cation, the nature and coverage ratio of the capping agent, and the polarity of the dispersing solvent [21]. In order to stabilize and maximize PL, a semiconductor shell with higher band gap not only passivates the surface bonds but also varies the semiconductor potential energy well, concentrating the charge carriers in a core away from the surface [22,23]. Thereby, surface defect states and trap sites will have a diminished impact on the PL efficiency, and fewer environmental factors will influence the emission intensity. At the beginning of a shell coating, the passivation of a surface led a quick increase of PL efficiency. When the shell grew to a desired thickness, the corresponding PL efficiency of the resulting core/shell QDs approached the maximum value.

**Figure 4:**
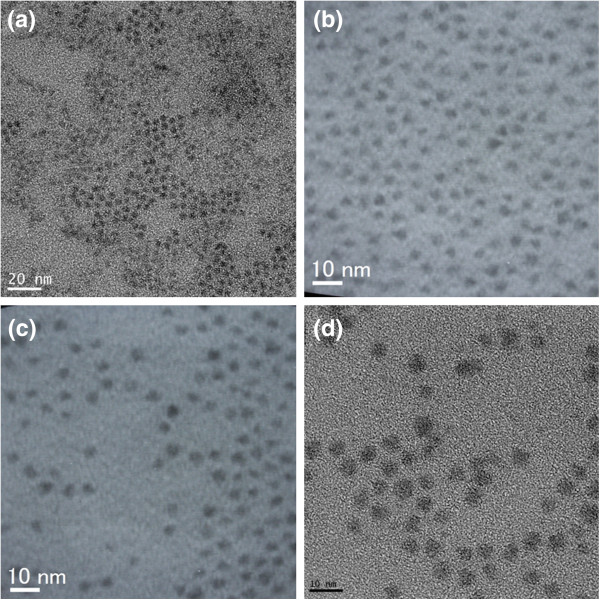
**TEM images of CdTe**_**0.5**_**Se**_**0.5**_**cores and CdTe**_**0.5**_**Se**_**0.5**_**/Cd**_**0.5**_**Zn**_**0.5**_**S core/shell QDs shown in Table**3**.** (**a**) Sample 5 (CdTe_0.5_Se_0.5_), (**b**) samples 5–4 (CdTe_0.5_Se_0.5_/Cd_0.5_Zn_0.5_S), (**c**) sample 5–8 (CdTe_0.5_Se_0.5_/Cd_0.5_Zn_0.5_S), and (**d**) sample 5–9 (CdTe_0.5_Se_0.5_/Cd_0.5_Zn_0.5_S). Samples 5–4, 5–8, and 5–9 were prepared by using sample 5.

To confirm the increase of shell thickness with time, Figure 4 shows the TEM images of CdTe_0.5_Se_0.5_ cores and CdTe_0.5_Se_0.5_/Cd_0.5_Zn_0.5_S core/shell QDs shown in Table 3: (a) sample 5 (CdTe_0.5_Se_0.5_), (b) sample 5–4 (CdTe_0.5_Se_0.5_/Cd_0.5_Zn_0.5_S), (c) sample 5–8 (CdTe_0.5_Se_0.5_/Cd_0.5_Zn_0.5_S), and (d) sample 5–9 (CdTe_0.5_Se_0.5_/Cd_0.5_Zn_0.5_S). The average diameter of samples 5, 5–4, 5–8, and 5–9 is 3.3, 3.6, 4.7, and 5.3 nm, respectively. The shell thickness of 1 nm resulted in a large red shift of 75.2 nm of the PL spectrum. This is significantly different from that of a CdSe core coated with a Cd_0.5_Zn_0.5_S shell [6]. This is ascribed to the effect of band gap of core and shell on PL properties.

**Figure 5:**
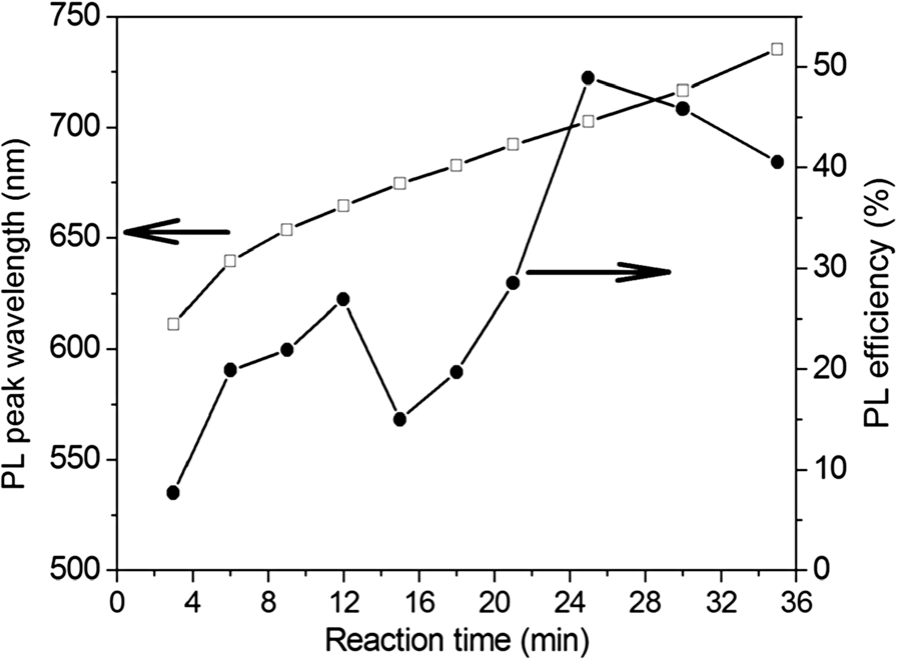
**PL peak wavelength and efficiency of CdTe**_**0.5**_**Se**_**0.5**_**/Cd**_**0.5**_**Zn**_**0.5**_**S core/shell QDs (Samples 6–1 to 6–10 were shown in Table**4**) versus reaction time.** The core/shell QDs were prepared by using sample 6. White square in the figure shows the PL peak wavelength, while black circle shows PL efficiency.

**Figure 6:**
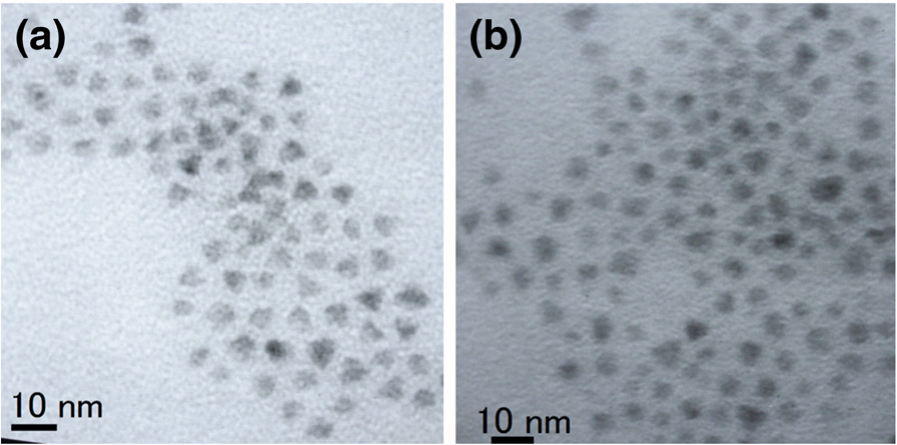
**TEM images CdTe**_**0.5**_**Se**_**0.5**_**/Cd**_**0.5**_**Zn**_**0.5**_**S core/shell QDs shown in Table**4**.** (**a**) Sample 6–5 and (**b**) sample 6–9. The core/shell QDs were prepared by using sample 6.

Because the size of CdTe_0.5_Se_0.5_ cores plays an important role for the PL properties of CdTe_0.5_Se_0.5_/Cd_0.5_Zn_0.5_S core/shell QDs, we fabricated the core/shell QDs using a large core. Table 4 summarizes the preparation condition and properties of CdTe_0.5_Se_0.5_/Cd_0.5_Zn_0.5_S QDs by a large CdTe_0.5_Se_0.5_ core (sample 6). The PL properties of sample 6 are listed for comparison. Figure 5 shows the PL peak wavelength and efficiency of CdTe_0.5_Se_0.5_/Cd_0.5_Zn_0.5_S core/shell QDs (samples shown in Table 4) versus reaction time. A gradual red shift of PL spectra was observed with time. The FWHM of PL spectra was increased after coating with a Cd_0.5_Zn_0.5_S shell. Compared with samples 5–9 shown in Table 3, samples 6–10 revealed a longer PL peak wavelength (735 nm) even though they were prepared using the same reflux time (35 min). This is ascribed to the increase in the core size. Because samples 6–10 exhibited a PL efficiency of 40%, the large CdTe_0.5_Se_0.5_ core can be a good candidate for the preparation of QDs with NIR and mid-infrared emission. Figure 6 shows the TEM images of CdTe_0.5_Se_0.5_/Cd_0.5_Zn_0.5_S core/shell QDs shown in Table 4: (a) sample 6–5 and (b) sample 6–9. Sample 6–5 revealed a much narrow size distribution than that of sample 6–9. Because a longer reflux time (sample 6–9) resulted in a broad size distribution as shown in Figure 6b, the FWHM of PL spectrum of sample 6–9 became large compared with that of sample 6–5 as shown in Table 4.

Generally, CdTe QDs show poor photostability because of the easy oxidation of tellurium as we have mentioned before. The stability was increased by being coated with a Zn_0.5_Cd_0.5_S shell. Especially in the case of CdTe_0.5_Se_0.5_ cores coated with a Cd_0.5_Zn_0.5_S shell, the core/shell QDs with a QD concentration of 100 nM in a toluene solution retained their initial PL efficiency for 1 month.

## Conclusions

A successful approach for preparing highly luminescent CdTe_0.5_Se_0.5_/Cd_0.5_Zn_0.5_S core/shell QDs has been presented. CdSe, CdTe, and CdTe_0.5_Se_0.5_ cores were first fabricated by a less toxic organic synthesis. Experimental results indicated that the size and PL properties depended strongly on the injection speed of anion precursors. CdSe and CdTe_0.5_Se_0.5_ cores exhibited high stability, while the PL of CdTe cores was quenched after 2 days. Subsequently, CdTe_0.5_Se_0.5_ cores were coated with Cd_0.5_Zn_0.5_S shells at relatively low temperature by using metal acetate and TOPS. This method has significant advantages: being less toxic, insensitive to air and moisture, easier to purify, and provide highly monodispersed QDs with a high PL efficiency and NIR emission. Namely, the Cd_0.5_Zn_0.5_S shell makes a CdTe_0.5_Se_0.5_ core a significant red shift of both absorption and PL spectra with reaction time. Experimental results indicated that the lower deposition temperature played a critical role for overgrowing shell material around the cores and getting high PL efficiency of the core/shell QDs. The emission wavelength from the core/shell QDs can span from the initial PL of the CdTe_0.5_Se_0.5_ core to 735 nm, which is achieved through the construction of the structure of a composite shell on a composite core. Because of tunable PL wavelength, high PL efficiency, and high stability, we expect that these core/shell QDs would be promising biomedical fluorophores for ultrasensitive, multicolor, and multiplexing applications, especially in *vivo* biomedical imaging applications.

## Competing interest

The authors declare that they have no competing interests.

## Authors’ contributions

PY carried out most of the experiment. SW measured the spectrum and analyzed the data. NM provided the research conditions and did the experiment and discussion together. All authors read and approved the final manuscript.
